# Control of Apoptosis by Asymmetric Cell Division

**DOI:** 10.1371/journal.pbio.0060084

**Published:** 2008-04-08

**Authors:** Julia Hatzold, Barbara Conradt

**Affiliations:** 1 Department of Genetics, Norris Cotton Cancer Center, Dartmouth Medical School, Hanover, New Hampshire, United States of America; 2 Max Planck Institute of Neurobiology, Planegg-Martinsried, Germany; University of Cambridge, United Kingdom

## Abstract

Asymmetric cell division and apoptosis (programmed cell death) are two fundamental processes that are important for the development and function of multicellular organisms. We have found that the processes of asymmetric cell division and apoptosis can be functionally linked. Specifically, we show that asymmetric cell division in the nematode Caenorhabditis elegans is mediated by a pathway involving three genes, *dnj-11* MIDA1, *ces-2* HLF, and *ces-1* Snail, that directly control the enzymatic machinery responsible for apoptosis. Interestingly, the MIDA1-like protein GlsA of the alga Volvox carteri, as well as the Snail-related proteins Snail, Escargot, and Worniu of Drosophila melanogaster, have previously been implicated in asymmetric cell division. Therefore, *C. elegans dnj-11* MIDA1, *ces-2* HLF, and *ces-1* Snail may be components of a pathway involved in asymmetric cell division that is conserved throughout the plant and animal kingdoms. Furthermore, based on our results, we propose that this pathway directly controls the apoptotic fate in C. elegans, and possibly other animals as well.

## Author Summary

Asymmetric cell division and apoptosis (programmed cell death) are two fundamental processes that are important for the development and function of multicellular organisms. Asymmetric cell division creates daughter cells of different fates, and this is critical for the generation of cellular diversity. Apoptosis eliminates superfluous cells from the organism, which is critical for cellular homeostasis. We found that the processes of asymmetric cell division and apoptosis can be functionally linked. Specifically, we show that asymmetric cell division in the nematode Caenorhabditis elegans is mediated by a pathway involving three genes, *dnj-11* MIDA1, *ces-2* HLF, and *ces-1* Snail, that directly control the enzymatic machinery responsible for apoptosis. Interestingly, the role of this pathway in asymmetric cell division and the control of apoptosis might be evolutionarily conserved. Furthermore, it might have an unexpected role in stem cell biology: the process of asymmetric cell division plays an essential role in the ability of stem cells to self-renew, and the mammalian counterparts of two components of the *dnj-11* MIDA1, *ces-2* HLF, *ces-1* Snail pathway have recently been implicated in stem cell function. For this reason, we speculate that a *dnj-11* MIDA1, *ces-2* HLF, *ces-1* Snail–like pathway might function in stem cells to coordinate self-renewal and apoptosis and, hence, the number of stem cells.

## Abstract 3

A pathway involved in asymmetric cell division in the nematode *Caenorhabditis elegans*, the *dnj-11* MIDA1, *ces-2* HLF, *ces-1* Snail pathway, directly controls the enzymatic machinery responsible for apoptosis.

## Introduction

Asymmetric cell division is essential for the generation of cellular diversity during animal development [1]. In certain cases, one of the cells derived from an asymmetric division is specified to undergo apoptosis (programmed cell death) [2–11]. However, the genetic and cell biological mechanisms that permit the coupling of asymmetric cell division and the adoption of the apoptotic fate are not well understood.

Previous studies have implicated members of the Snail family of transcriptional repressors in both asymmetric cell division and apoptosis, but there has been no demonstration that these processes are integrated in a given developmental context. In mammals, Snail-related proteins have been shown to have pro-survival as well as anti-apoptotic activities, and they have been causally linked to tumorigenesis and tumor progression in mammals [12,13]. In Drosophila melanogaster, the Snail-related genes *snail*, *escargot*, and *worniu* have been shown to function redundantly in asymmetric cell division [14,15]. Specifically, rather than dividing asymmetrically and producing two daughter cells of different sizes and fates, neuroblasts in the central nervous system of D. melanogaster mutants lacking *snail*, *escargot*, and *worniu* function divide symmetrically to produce two daughter cells of similar sizes and fates. The effect of *snail*, *escargot*, and *worniu* on asymmetric neuroblast division is mediated in part by their ability to promote the expression of the gene *inscuteable*, which encodes an adaptor protein that is required for the establishment and maintenance of neuroblast polarity [16]. The protein Inscuteable is localized in a polar fashion through its interaction with a complex composed of the PDZ domain–containing proteins Bazooka (also referred to as Par-3) and Par-6, which are found on the apical cell cortex of the neuroblasts [17]. Inscuteable in turn recruits the adaptor protein Pins and the alpha subunit of the heterotrimeric G protein Gi (Gαi), which initiates the displacement of the mitotic spindle along the cell division axis of the neuroblasts, resulting in their asymmetric division. Inscuteable is also at least partially required for the enrichment of cell-fate determinants such as Prospero and Staufen on the basal cell cortex of the neuroblasts and their asymmetric segregation into the basal daughter cell [17].

**Figure 1:**
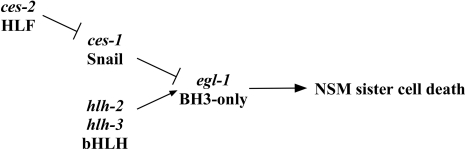
The Genetic Pathway of the NSM Sister Cell Death *egl-1* is required for the death of the NSM sister cell. *egl-1* activity in the NSM sister cell is positively regulated by the genes *hlh-2* and *hlh-3* and negatively regulated by the gene *ces-1*. The activity of *ces-1* is negatively regulated by the gene *ces-2*. See text for details.

In Caenorhabditis elegans, the Snail-related protein CES-1 (cell-death specification) has been implicated in the suppression of a specific apoptotic event, the death of the neurosecretory motoneuron (NSM) sister cell [18,19]. During embryonic development, the NSM neuroblast divides to give rise to two daughter cells: the NSM, which differentiates into a serotonergic motoneuron, and the NSM sister cell, which undergoes apoptosis [3]. A gain-of-function mutation of the *ces-1* gene, which most likely results in the mis- or overexpression of *ces-1* in the NSM lineage, prevents the death of the NSM sister cell [18]. The death of the NSM sister cell is dependent on the transcriptional upregulation of the pro-apoptotic BH3-only gene *egl-1* (egg-laying abnormal) in the NSM sister cell, a process that is at least partially dependent on a heterodimer composed of the bHLH transcription factors HLH-2 and HLH-3 (HLH-2/HLH-3) [20] (Figure 1). HLH-2/HLH-3 can bind to E-boxes/Snail-binding sites in a *cis*-regulatory region of the *egl-1* locus referred to as Region B, which is required for the expression of *egl-1* in the NSM sister cell in vivo. Therefore, it has been proposed that HLH-2/HLH-3 is a direct activator of *egl-1* transcription in the NSM sister cell. The *ces-1*(gf) mutation prevents the death of the NSM sister cell by blocking the HLH-2/HLH-3–dependent expression of *egl-1* in the NSM sister cell [18,20]. Furthermore, CES-1 can also bind to the E-boxes/Snail-binding sites in Region B of the *egl-1* locus, and the ability of elevated levels of CES-1 to prevent the death of the NSM sister cell in vivo is dependent on these E-boxes/Snail-binding sites. On the basis of these observations, it has been proposed that by competing with HLH-2/HLH-3 for binding to Region B of the *egl-1* locus, elevated levels of CES-1 protein in *ces-1*(gf) animals directly block *egl-1* transcription in the NSM sister cell [20]. Finally, it has been hypothesized that the CES-1 protein might normally function in the NSM to block HLH-2/HLH-3–dependent *egl-1* transcription, thereby allowing the survival of the NSM.

The function of the *ces-1* gene in the NSM sister cell is negatively regulated by the gene *ces-2*, which is required for the death of the NSM sister cell and which encodes a bZIP transcription factor similar to the human proto-oncoprotein HLF (hepatic leukemia factor) [18,21] (Figure 1). Here we report that the previously uncharacterized protein DNJ-11 (DnaJ domain–11), a MIDA1 (mouse Id associated 1)–like chaperone, cooperates with the CES-2 protein to reduce *ces-1* transcription in the NSM lineage, thereby excluding CES-1 protein from the NSM sister cell and allowing the death of the NSM sister cell. Furthermore, we show that the NSM neuroblast, which gives rise to the NSM and NSM sister cell, divides asymmetrically and that the genes *dnj-11*, *ces-2*, and *ces-1* also function to cause asymmetric NSM neuroblast division. Our results reveal new developmental roles of MIDA1-like chaperones and HLF-like transcription factors. Furthermore, our results delineate a pathway involved in asymmetric cell division that directly controls the apoptotic fate.

## Results

### *bc212* Prevents the Death of the NSM Sister Cells

The NSM sister cells are generated about 410 min after the first cleavage of the C. elegans zygote and undergo apoptosis at about 430 min [3]. We screened for mutations that cause the NSM sister cells to survive, and we isolated the recessive mutation *bc212* (J. Hatzold, B. Conradt, unpublished data). *bc212* causes 12% and 50% NSM sister cell survival when raised at 25 °C or 15 °C, respectively, and hence causes a cold-sensitive NSM sister cell survival phenotype (Table 1, *dnj-11(bc212)*). In addition, *bc212* is maternally rescued: in homozygous *bc212* progeny (*dnj-11(bc212)*) of heterozygous *bc212* hermaphrodites (*dnj-11(bc212)*/+), only 1% of the NSM sister cells survive (Table S1). To determine whether *bc212* prevents the death of cells other than the NSM sister cells, we analyzed additional cells that undergo apoptosis during C. elegans development. We found that *bc212* had no effect on their deaths, which demonstrates that *bc212* does not block apoptosis in general (Table S2). However, we found that *bc212* causes a variety of other defects such as morphological defects, lethality, slow growth, and reduced brood size (Figure S1 and Tables S3–S5). Therefore, the gene defined by *bc212* is required for the death of the NSM sister cells and for additional processes that are important for C. elegans development and fertility.

**Table 1:**
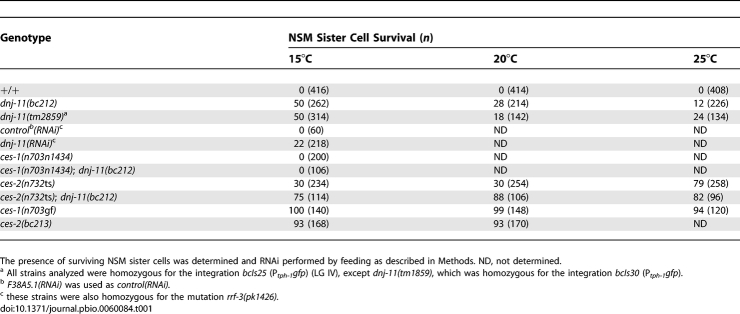
*bc212* Causes a Cold-Sensitive NSM Sister Cell Survival Phenotype.

### *bc212* Is a Putative Null Allele of the Gene *dnj-11*, Which Is Widely Expressed and Encodes a Protein That Primarily Localizes to the Cytosol

**Figure 2:**
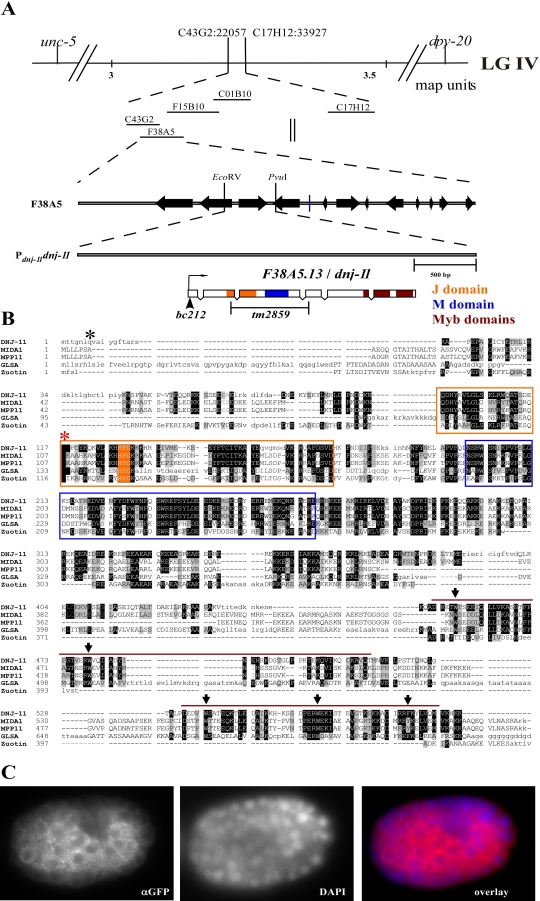
*dnj-11* Encodes a Member of the Family of MIDA1-Like Proteins (A) (Top) Genes and single-nucleotide polymorphisms (SNPs) used for mapping *bc212* are indicated. (Middle) Cosmids assayed for rescue of *dnj-11(bc212)* are shown. (Bottom) P*_dnj-11_dnj-11* is a subclone of *F38A5* and represents the minimal *dnj-11(bc212)* rescue fragment. The J domain, M domain, and Myb domains of the *dnj-11* ORF are indicated in orange, blue, and maroon, respectively. (B) The alignment of the DNJ-11 sequence with the sequence of mouse MIDA1, human MPP11, V. cateri GlsA, and the S. cerevisiae Zuotin was performed using the DIALIGN algorithm. Identical amino acids and similar amino acids are shaded in black and gray, respectively. The orange box marks the J domain and the conserved tripeptide is shaded in orange. The blue box indicates the M domain. The maroon bars above the sequences represent the Myb domains. Arrows point to conserved aromatic residues in the Myb domains. *bc212* results in the truncation of the DNJ-11 protein after aa 6 (asterisk). *tm2859* results in a frame shift after aa 116 (red asterisk) and a stop after an additional 67 aas in a different frame. (C) Embryos transgenic for the functional P*_dnj-11_dnj-11*::*gfp* transgene were fixed as described in Materials and Methods and stained with an anti-GFP antibody (αGFP) to detect DNJ-11::GFP and with DAPI (DAPI) to detect nuclei.

We cloned the gene defined by *bc212* and found that it is identical to the previously uncharacterized gene *dnj-11* (*F38A5.13*) (DNJ-11 accession number NP_501006) on linkage group (LG) IV (Figure 2A). *dnj-11* encodes a 589–amino acid (aa) protein that is most similar to members of the family of MIDA1-like proteins, which are found in plants and animals (see below). *bc212* is a C-to-T transition at position 21 of the nucleotide sequence of the *dnj-11* gene and transforms codon 7 into a stop codon, which is predicted to truncate the DNJ-11 protein after aa 6 (Figure 2A and 2B). *tm2859*, a 641–base pair (bp) deletion of the *dnj-11* gene, removes base pairs 402-1042 of the *dnj-11* coding region. This deletion is predicted to result in a frameshift leading to the generation of a truncated protein composed of the first 116 aa of the wild-type protein and a 67-aa, C-terminal extension in a different reading frame (Figure 2A and 2B). Like *bc212*, *tm2859* causes a cold-sensitive NSM sister cell survival phenotype and 50% NSM sister cell survival at 15 °C (Table 1, *dnj-11(tm2859)*). In addition, *bc212* and *tm2859* cause 24% and 26% lethality, respectively, at 15 °C (Table S3). Based on these results, we conclude that *bc212* represents a strong loss-of-function mutation and putative null allele of the *dnj-11* gene.

Using a functional transgene that drives the expression of a DNJ-11::green fluorescent protein (GFP) fusion protein under the control of the *dnj-11* promoter (P*_dnj-11_dnj-11*::*gfp*), we determined the expression pattern of the *dnj-11* gene and the sub-cellular localization of the DNJ-11 protein. P*_dnj-11_dnj-11*::*gfp* expression was observed in most if not all somatic cells of embryos, larvae, and adult animals, including the cells of the NSM lineage (Figure 2C and unpublished data). DNJ-11::GFP protein primarily localized to the cytoplasm in a punctate pattern and could not be detected in nuclei. In addition, the *dnj-11* gene is most likely also expressed in the adult germ line, because *dnj-11(bc212)* is maternally rescued (Table S1).

### *dnj-11* Cooperates with *ces-2* to Negatively Regulate *ces-1*

The death of the NSM sister cells is dependent on the transcriptional upregulation in the NSM sister cells of the BH3-only gene *egl-1* (EGL-1 accession number NP_506575) [20]. The transcriptional upregulation of *egl-1* in the NSM sister cells can be blocked by the Snail-related transcription factor CES-1 (accession number NP_492338), which in turn is negatively regulated by the HLF-like transcription factor CES-2 (accession number NP_493610) [18–21]. By using an *egl-1* transgene (P*_egl-1_his-24*::*gfp*), we found that *dnj-11(bc212)* resulted in the loss of *egl-1* expression in 69% of the NSM sister cells, indicating that *dnj-11* acts upstream of *egl-1* to promote its transcription in the NSM sister cells (Table S6). To determine where *dnj-11* functions with respect to the genes *ces-1* and *ces-2*, we analyzed the interactions of *dnj-11(bc212)* with a putative null mutation of *ces-1—* *n703n1434* [19]*—*and a partial, temperature-sensitive loss-of-function (lf) mutation of *ces-2—n732* [18]. We found that 0% of the NSM sister cells survived in *ces-1(n703n1434)*; *dnj-11(bc212)* double mutants, demonstrating that the ability of *dnj-11(bc212)* to prevent the death of the NSM sister cells requires a functional *ces-1* gene (Table 1, *ces-1(n703n1434)*; *dnj-11(bc212)*). Finally, we found that at 20 °C, *dnj-11(bc212)* greatly enhanced the NSM sister cell survival phenotype caused by *ces-2(n732*) (Table 1, *ces-2(n732)*; *dnj-11(bc212)*). These results indicate that, at least in the NSM lineage, *dnj-11* acts upstream of and as a negative regulator of *ces-1*. Furthermore, these results suggest that *dnj-11* and *ces-2* cooperate to antagonize *ces-1* function.

### The J Domain and Myb Domains of the MIDA1-like DNJ-11 Protein Are Required for Its Function

MIDA1-like proteins contain three major regions: (1) an N-terminal J domain, which is a protein–protein interaction domain found in members of the J protein or Hsp40 family of chaperones [22], (2) a central M domain, which is another protein–protein interaction domain found specifically in MIDA1-like proteins [23], and (3) two C-terminal Myb domains, which are DNA-binding domains typically found in transcription factors [24,25] (Figure 1A and 1B). MIDA1-like proteins have mainly been implicated in growth control [23,26,27] and are thought to function by regulating transcription and translation [28–32]. BLAST searches [33] revealed that the DNJ-11 protein is highly similar to mouse MIDA1 (40% identical, 61% similar) (accession number NP_033610) [23], human MPP11 (M phase phosphoprotein) (38% identical, 54% similar) (accession number NP_055192) [27], Volvox carteri GlsA (gonidialess) (34% identical, 52% similar) (accession number AF_106963 GenBank) [26], and Saccharomyces cerevisiae Zuotin (37% identical, 62% similar) (accession number NP_011801) [34] (Figure 1B).

To determine whether the J domain is important for DNJ-11 activity, we replaced the histidine residue of the conserved tripeptide of the J domain with a glutamine residue (H129Q) and tested a transgene that expresses a DNJ-11(H129Q)::GFP fusion protein under the control of the *dnj-11* promoter (P*_dnj-11_dnj-11*(H129Q)::*gfp*) for its ability to rescue the NSM sister cell survival phenotype caused by *dnj-11(bc212)*. In contrast to P*_dnj-11_dnj-11*::*gfp*, P*_dnj-11_dnj-11*(H129Q)::*gfp* failed to rescue the NSM sister cell survival phenotype of *dnj-11*(*bc212*) animals (Table 2). To determine whether the Myb domains are important for DNJ-11 activity, we replaced the tryptophan residue at position 456 in the first Myb domain (W456) and/or the phenylalanine residue at position 578 in the second Myb domain (F578) with glycine and tested the resulting transgenes (P*_dnj-11_dnj-11*(W456G)::*gfp*, P*_dnj-11_dnj-11*(F578G)::*gfp*, and P*_dnj-11_dnj-11*(W456G F578G)::*gfp*) for their ability to rescue the NSM sister cell survival phenotype caused by *dnj-11(bc212)*. We found that the transgenes expressing a DNJ-11::GFP fusion protein with either one of the Myb domains mutated partially rescued the NSM sister cell survival phenotype of *dnj-11(bc212)* animals (Table 2, *dnj-11(bc212)*; P*_dnj-11_dnj-11*(W456G)::*gfp*, *dnj-11(bc212)*; P*_dnj-11_dnj-11*(F578G)::*gfp*). However, the transgene expressing a DNJ-11::GFP fusion protein with both Myb domains mutated was no longer able to rescue (Table 2, *dnj-11(bc212)*; P*_dnj-11_dnj-11*(W456G F578G)::*gfp*). These results demonstrate that the J domain and at least one functional Myb domain are required for the ability of DNJ-11 to cause the death of the NSM sister cells.

**Table 2:**
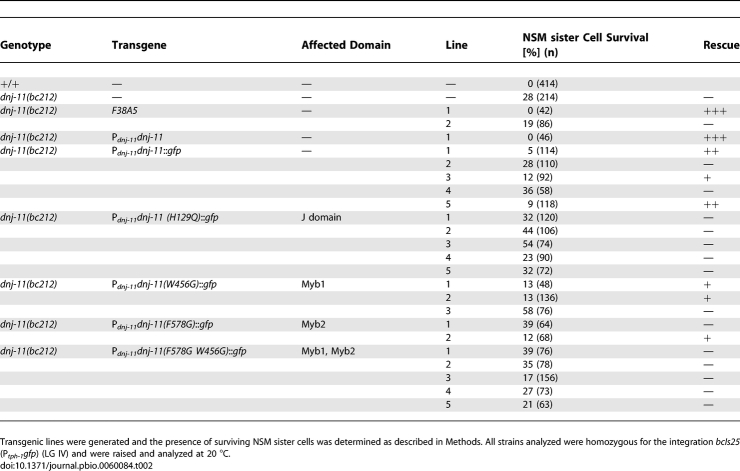
The J Domain and the MYB Domains Are Required for the Ability of DNJ-11 to Cause the Death of the NSM Sister Cells

### *dnj-11* Is Required for Asymmetric NSM Neuroblast Division

The V. carteri ortholog of DNJ-11, GlsA, has been implicated in asymmetric cell division [35]. Specifically, during V. carteri development, an asymmetric cell division occurs that results in the generation of two daughter cells of different sizes and fates, namely a large reproductive cell and a small somatic cell. In *glsA* mutants, this cell division occurs symmetrically, resulting in two cells of equal size, both of which differentiate into somatic cells [26].

**Figure 3:**
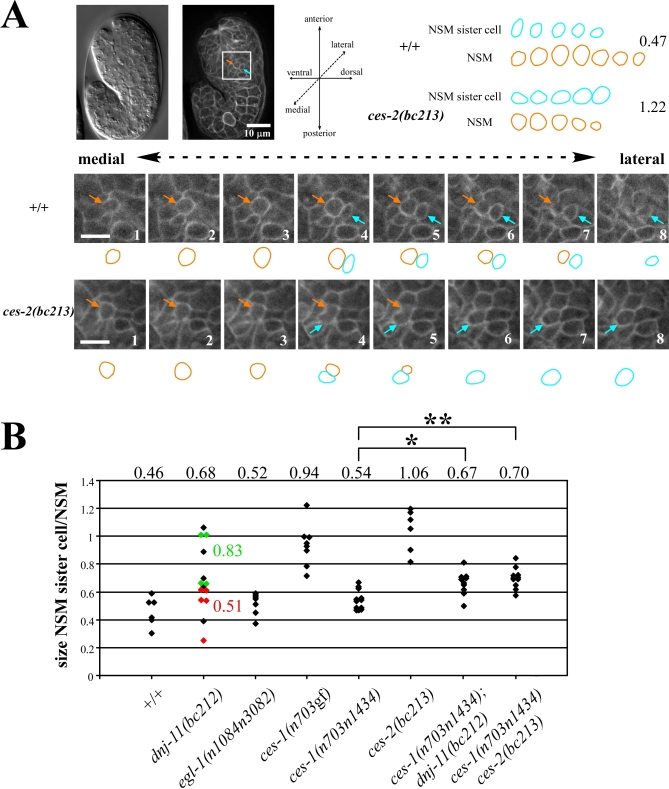
*dnj-11*, *ces-1*, and *ces-2* Are Involved in Asymmetric NSM Neuroblast Division (A) (Left top) Nomarski and epifluorescence image of a wild-type embryo transgenic for the transgene P*_pie-1_gfp*::*ph*(*PLC1*δ*1)* immediately after the NSM neuroblast divided. The orange arrow points to the NSM, the blue arrow points to the NSM sister cell. (Bottom) Series of eight consecutive focal planes (0.5 μm distance) (1–8) through the NSM and NSM sister cell of a wild-type (upper panel, +/+) or *ces-2(bc213*) (lower panel, *ces-2(bc213*)) embryo. (Right top) Summary of schematic representations of the outlines. Numbers on the right indicate the ratios between the sizes of the NSM sister cells and the NSMs in the Z-series shown. (B) The ratio of the sizes of the NSM sister cell and the NSM was determined as described in Materials and Methods. The NSM sister cell was defined as the lateral cell of the two daughter cells. Each diamond represents the ratio of a single, independent NSM neuroblast division. The numbers above the diamonds represent the average ratio obtained for a given genotype. The apoptotic fate of the NSM sister cells was determined in a subset of *dnj-11(bc212)* embryos (red/died, green/survived). The complete genotypes of the embryos analyzed from left to right were: *tIs38* (P*_pie-1_gfp*::*ph*(*PLC1δ1)*), *dnj-11(bc212) bcIs25*; *tIs38*, *egl-1(n1048n3082)*; *tIs38*, *ces-1(n703*gf*)*; *tIs38*, *ces-1(n703n1434)*; *tIs38*, *ces-2(bc213)*; *bcIs25*; *tIs38*, *ces-1(n703n1434)*; *dnj-11(bc212) bcIs25*; *tIs38*, and *ces-1(n703n1434) ces-2(bc213)*; *bcIs25*; *tIs38*. *p*-Values were determined by the Student's *t*-test. *ces-1(n703n1434) compared to *ces-1(n703n1434)*; *dnj-11(bc212)*: p<0.002, **ces-1(n703n1434) compared to *ces-1(n703n1434) ces-2(bc213)*: p<0.001.

Most of the 131 cells that undergo apoptosis during C. elegans development are thought to be the result of an asymmetric cell division that gives rise to a large cell, which is programmed to survive, and a small cell, which is programmed to die [2,3]. Therefore, we determined whether the NSM neuroblast, which gives rise to the NSM and the NSM sister cell, divides asymmetrically with respect to size as well. To that end, we identified the NSM neuroblast based on its position in the embryo, observed its division at about 410 min, and determined the sizes of its daughter cells immediately after its division had been completed (Figure 3A). Using a transgene that expresses a plasma membrane–targeted GFP fusion protein (P*_pie-1_gfp*::*ph*(*PLC1*δ*1)*) [36], we found that in wild-type animals, the size of the NSM sister cell on average is 0.46 times the size of the NSM (Figure 3B, +/+). Therefore, the NSM neuroblast divides asymmetrically to give rise to a large cell (the NSM) and a small cell (the NSM sister cell).

Furthermore, we found that in *dnj-11*(*bc212)* animals, the difference in size between the NSM sister cell and the NSM is highly variable, ranging from a 2-fold difference as observed in wild-type animals to no difference (Figure 3B, *dnj-11*(*bc212)*). To determine whether the range observed reflects the incomplete penetrance of the NSM sister cell survival phenotype caused by *dnj-11(bc212)* (see Table 1), we followed the fate of NSM sister cells after the division of the NSM neuroblasts. We found that in five out of five embryos in which the division of the NSM neuroblast had occurred asymmetrically (NSM sister cell size on average 0.51 times the size of the NSM), the NSM sister cells died (Figure 3B, red diamonds). By contrast, four out of four NSM sister cells that were similar in size to the NSMs (NSM sister cell size on average 0.83 times the size of the NSM) survived (green diamonds). To rule out the possibility that the increase in NSM sister cell size observed in *dnj-11(bc212)* animals is a result of inappropriate NSM sister cell survival rather than a defect in asymmetric cell division, we analyzed *egl-1*(lf) mutants, in which many apoptotic events, including the death of the NSM sister cells, are blocked. We found that, like in wild-type animals, the NSM neuroblast divided asymmetrically in *egl-1*(lf) mutants (NSM sister cell size on average 0.52 times the size of the NSM) (Figure 3B, *egl-1(n1084n3082)*). Thus, the increase in NSM sister cell size observed in *dnj-11(bc212)* animals is a result of a defect in asymmetric cell division. Therefore, we conclude that *dnj-11* is required for asymmetric NSM neuroblast division and, by inference, is required for the displacement of the mitotic spindle along the cell division axis. Furthermore, because in *dnj-11(bc212)* animals the defect in asymmetric NSM neuroblast division correlates with the defect in NSM sister cell death, asymmetric NSM neuroblast division might be critical for the specification of the apoptotic fate of the NSM sister cell.

To determine whether *dnj-11* is required for asymmetric cell division in lineages other than the NSM lineage, we analyzed a subset of the other asymmetric cell divisions (including the first division of the zygote) that take place during C. elegans development and that give rise to daughter cells of different sizes and fates. We found that none of these divisions was affected by *dnj-11(bc212)*, suggesting that *dnj-11* is not required for asymmetric cell division in general (Table S7 and unpublished data).

### *ces-1* and *ces-2* also Function in Asymmetric NSM Neuroblast Division

Like *dnj-11(bc212)*, the *ces-1*(gf) mutation *n703* and a putative null mutation of *ces-2*, *bc213*, prevent the death of the NSM sister cells (Table 1, *ces-1*(*n703*gf), *ces-2*(*bc213*)). Unexpectedly, we found that *ces-1*(*n703*gf) and *ces-2*(*bc213*) also disrupt the asymmetric division of the NSM neuroblast and result in the production of two cells of similar sizes (NSM sister cell sizes on average 0.94 and 1.06 times the size of the NSM) (Figure 3B, *ces-1(n703*gf), *ces-2*(*bc213*)). These results demonstrate that increased levels of *ces-1* function can prevent asymmetric NSM neuroblast division and that, like *dnj-11*, *ces-2* is required for asymmetric NSM neuroblast division.

To determine whether *ces-1* also acts downstream of *dnj-11* and *ces-2* during asymmetric NSM neuroblast division, we determined whether the loss of *ces-1* function can suppress the defects in asymmetric NSM neuroblast division observed in *dnj-11(bc212)* and *ces-2(bc213)* animals. We found that *ces-1(n703n1434)* partially suppressed the defects in asymmetric NSM neuroblast division that are observed in *dnj-11(bc212)* and *ces-2(bc213)* animals (Figure 3B). This finding suggests that *ces-1* acts downstream of *dnj-11* and *ces-2* during asymmetric NSM neuroblast division as well.

### *dnj-11*, *ces-2*, and *ces-1* Also Play a Role in Establishing the Correct Orientation of the Cell Division Axis During Asymmetric NSM Neuroblast Division

Shortly after the division of the NSM neuroblast has been completed, the NSM is located at a position that is medial and ventral to the NSM sister cell (Figure 3A). The position of the NSM relative to the NSM sister cell implies that in wild-type animals, the cleavage plane of the NSM neuroblast is along the ventral/lateral to dorsal/medial axis. While determining the size of the NSM and NSM sister cell using the plasma membrane–targeted GFP fusion protein, we observed that in two out of nine *ces-2(bc213)* animals, the medial daughter cell was located dorsally rather than ventrally to the lateral daughter cell (Figure 3A). This observation suggests that the loss of *ces-2* function not only affects the displacement of the mitotic spindle along the cell division axis in the NSM neuroblast, but also the orientation of the cell division axis and, hence, the orientation of the cleavage plane.

**Figure 4:**
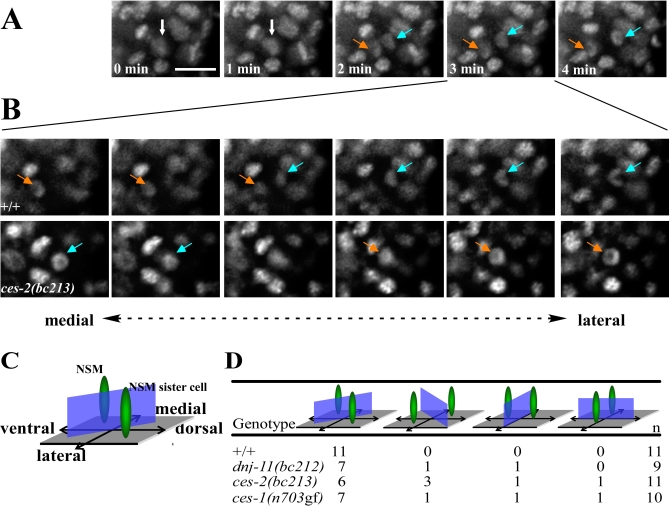
*dnj-11*, *ces-1*, and *ces-2* Are Involved in Orienting the Mitotic Spindle of the NSM Neuroblasts (A) Epifluorescent images shown are maximum projections of Z-series of a wild-type embryo carrying the transgene P*_his-24_his-24*::*gfp*. The scale bar represents 5 μm. Ventral is to the left and dorsal to the right. The Z-series were taken every 60 s during a 4-min time period. Chromosomal DNA in the NSM neuroblast prior to the division is indicated by white arrows. Between minute 1 and minute 2, the NSM neuroblast starts dividing. The chromatids of the NSM (indicated by orange arrows) move toward the ventral side, the chromatids of the NSM sister cell (indicated by blue arrows) move to the dorsal side. (B) (Upper panel). Epifluorescence images of six consecutive focal planes of the Z-series at minute 3 shown in (A) as maximum projection. Medial to lateral planes are shown from left to right. The chromatids of the ventral NSM are located medially. The chromatids of the dorsal NSM sister cell are located laterally. (Lower panel). Six consecutive focal planes of a Z-series of a *ces-2(bc213)* embryo. Medial to lateral planes are shown from left to right. The chromatids of the ventral NSM are located laterally. The chromatids of the dorsal NSM sister cell are located medially. (C) Schematic representation of the ventral/dorsal and medial/lateral positions of the chromatids of NSM and NSM sister cell (indicated in green) in wild-type embryos. The blue rectangle indicates the cleavage plane of the NSM neuroblast. (D) Quantification of the orientation of the cleavage planes observed. The complete genotypes of the embryos analyzed from top to bottom were: *dtIs372*; *dnj-11(bc212) bcls25; dtls372, ces-2(bc213); bcls25; dtls372*, and *ces-1(n703)*; *bcIs25*; *dtIs372*.

To observe more directly the division of the NSM neuroblast, we used a transgene that expresses a DNA-targeted GFP fusion protein (P*_his-24_his-24*::*gfp*) (M. Dunn, G. Seydoux, J. Waddle, personal communication). This fusion protein allowed us to determine the axis along which the separated chromatids move during anaphase of the NSM neuroblast division and therefore the cleavage plane of the dividing NSM neuroblast. In wild-type embryos, the chromatids of the future NSM move to the ventral/medial side, whereas the chromatids of the future NSM sister cell move to the dorsal/lateral side, confirming that the cleavage plane of the NSM neuroblast is along the ventral/lateral to dorsal/medial axis (Figure 4 ; +/+). As in wild-type embryos, we found that in *ces-2(bc213)* embryos, the cleavage plane in six out of 11 NSM neuroblasts was along the ventral/lateral to dorsal/medial axis. In three cases, however, the cleavage plane was reversed, and the cells divided along the ventral/medial to dorsal/lateral axis. Furthermore, in two cases, the cleavage plane was either along the lateral to medial or ventral to dorsal axis (Figure 4B and 4D; *ces-2(bc213)*). We next analyzed *dnj-11*(*bc212*) and *ces-1*(*n703*gf) embryos and found that the orientation of the cleavage plane of the NSM neuroblast was disrupted in two out of nine and three out of 10 NSM neuroblasts, respectively (Figure 3D; *dnj-11*(*bc212*), *ces-1*(*n703*gf)). Based on these findings, we conclude that *dnj-11*, *ces-2*, and *ces-1* not only play a role in the displacement of the mitotic spindle along the cell division axis in the NSM neuroblast but also in the orientation of the cell division axis.

### *dnj-11* and *ces-2* Reduce *ces-1* Transcription in the NSM Lineage Thereby Restricting the Presence of CES-1 Protein to the NSM

**Figure 5:**
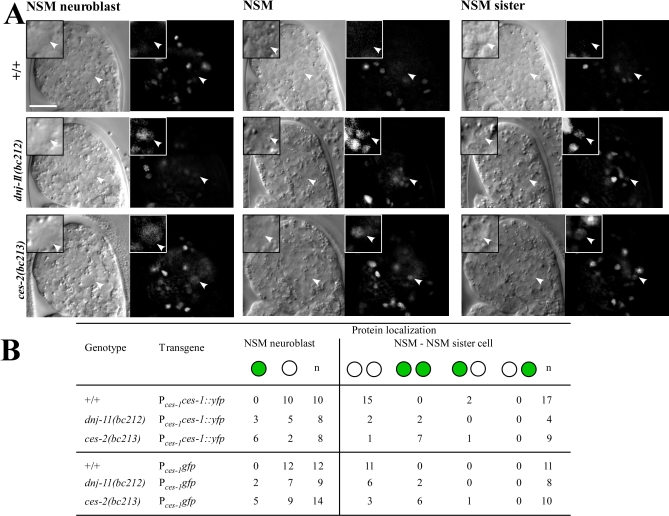
The Loss of *dnj-11* or *ces-2* Function Results in Increased Levels of CES-1 Protein and Increased *ces-1* Transcription in the NSM Lineage (A) Nomarski and epifluorescence images of wild-type (+/+), *dnj-11(bc212)*, and *ces-2(bc213)* embryos transgenic for a stable P*_ces-1_ces-1*::*yfp* transgene. White arrowheads indicate NSM neuroblasts (left column), NSMs (middle column), and NSM sister cells (right column). The scale bar represents 10 μm. Insets in the upper left corner of each image show the respective cells at a higher magnification. (B) Summary of the data on the presence of CES-1 protein in the NSM lineage using the P*_ces-1_ces-1*::*yfp* transgene and *ces-1* expression using the P*_ces-1_gfp* transgene. Green circles represent cells in which CES-1::YFP or GFP were detected. White circles represent cells in which CES-1::YFP or GFP were not detected. The complete genotypes of the embryos analyzed from top to bottom were: *bcIs58* (P*_ces-1_ces-1*::*yfp*), *dnj-11(bc212) bcIs25*; *bcIs58*, *ces-2(bc213)*; *bcIs25*; *bcIs58*, *bcEx619* (P*_ces-1_gfp*), *dnj-11(bc212) bcIs25*; *bcEx619*, and *ces-2(bc213)*; *bcIs25*; *bcEx619*.

To investigate how *dnj-11* and *ces-2* antagonize *ces-1* function in the NSM lineage, we constructed a functional transgene that drives the expression of a CES-1::yellow fluorescent protein (YFP) fusion protein under the control of the *ces-1* promoter (P*_ces-1_ces-1*::*yfp*). In wild-type animals, we failed to detect CES-1::YFP in NSM neuroblasts or NSM sister cells. However, we observed CES-1::YFP in two out of 17 NSMs analyzed (Figure 5A and 5B, +/+). (CES-1::YFP was observed in lineages other than the NSM lineage in all animals examined.) This observation suggests that the level of CES-1::YFP and hence, most probably of endogenous CES-1 protein, is relatively low in the NSM lineage and that it is higher in NSMs than in NSM neuroblasts or NSM sister cells. In *dnj-11(bc212)* and *ces-2(bc213)* animals, we observed CES-1::YFP not only in NSMs but also in NSM sister cells and NSM neuroblasts (Figure 5A and 5B, *dnj-11(bc212)*, *ces-2(bc213)*). These results demonstrate that *dnj-11* and *ces-2* antagonize *ces-1* function in the NSM lineage by reducing the level of CES-1 protein in the NSM neuroblast, thereby restricting the presence of CES-1 protein to the NSM.

To determine whether *dnj-11* and *ces-2* act at the transcriptional or posttranscriptional level to affect the level of CES-1 protein, we constructed a transgene that drives the expression of the GFP protein under the control of the *ces-1* promoter (P*_ces-1_gfp*) and analyzed GFP expression in the NSM lineage. In wild-type animals, GFP was not detected in NSM neuroblasts, NSMs, or NSM sister cells (Figure 5B, +/+). However, in *dnj-11(bc212)* and *ces-2(bc213)* animals, GFP was detected in NSM neuroblasts, NSMs, and NSM sister cells (Figure 5B, *dnj-11(bc212)*, *ces-2(bc213)*). Based on these results, we conclude that *dnj-11* and *ces-2* restrict the presence of CES-1 protein to the NSM by reducing *ces-1* transcription in the NSM lineage.

## Discussion

It is well established that asymmetric cell division can be coupled to the apoptotic fate [2–11]. However, the genetic and mechanistic basis for this relationship has not been well defined. Here we present evidence that the regulation of asymmetric cell division and apoptosis are functionally linked by a pathway that involves three evolutionarily conserved genes: *dnj-11* MIDA1, *ces-2* HLF and *ces-1* Snail.

### CES-1 Snail Levels Determine the Apoptotic Fate

**Figure 6:**
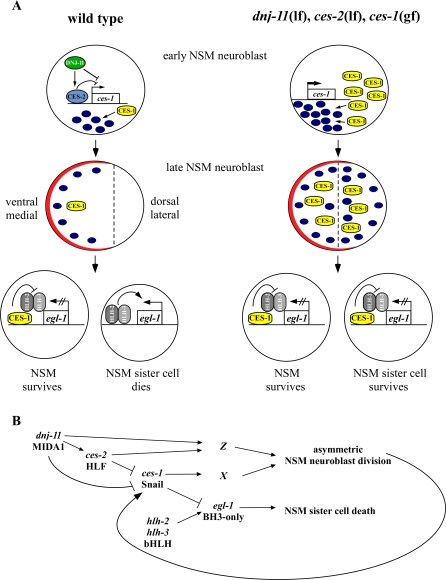
Regulation of Asymmetric NSM Neuroblast Division and the Apoptotic Death of the NSM sister Cell (A) Molecular models. (Wild type) By repressing the transcription of the *Snail*-related gene *ces-1*, the proteins CES-2 HLF and DNJ-11 MIDA1 ensure that the CES-1 Snail protein is present at an appropriate, low level in the unpolarized, early NSM neuroblast. Blue oval indicates polarity factor that is required to establish polarity in the late NSM neuroblast and the synthesis of which is promoted by CES-1. Indicated in red is a complex that restricts the polarity factor to the ventral/medial side of the NSM neuroblast. Once localized to the ventral/medial side, the polarity factor is involved in restricting CES-1 to the ventral/medial side of the late NSM neuroblast. See text for details. (*dnj-11*(lf), *ces-2*(lf), *ces-1*(gf)) Loss-of-function mutations of *dnj-11* or *ces-2*, or a gain-of-function mutation of *ces-1* result in an increased level of CES-1 Snail protein in the early NSM neuroblast. This increased level of CES-1 protein results in a level of polarity factor too high to establish asymmetry and restrict CES-1 protein to the ventral/medial side. See text for details. (B) Genetic pathway. *dnj-11* acts upstream of or in parallel to *ces-2* to negatively regulate the function of *ces-1*, thereby causing asymmetric NSM neuroblast division and NSM sister cell death. *ces-1* function can affect asymmetric NSM neuroblast division by regulating the function of a gene or genes required for the process. By negatively regulating *egl-1*, *ces-1* function can also prevent the *hlh-2*, *hlh-3–*dependent death of the NSM sister cells. *ces-1* function in NSM sister cell death is affected by the process of asymmetric NSM neuroblast division, which causes the asymmetric distribution of the product of the *ces-1* gene. See text for details.

Snail family transcription factors have previously been implicated in the regulation of apoptosis in mammalian cells. In the case of C. elegans, it has been suggested that the Snail protein, CES-1, might normally function within the NSM lineage to repress transcription of the pro-apoptotic gene, *egl-1*, thereby allowing NSM survival. Our data support this idea, because we find that CES-1 protein is present at higher levels in the NSMs than in the NSM sister cells. Therefore, we propose that CES-1 acts as a cell-fate determinant in the NSMs to ensure the survival of the NSMs and their differentiation into serotonergic neurons. Conversely, we propose that the absence of CES-1 protein in the NSM sister cells determines the death of the NSM sister cells (Figure 6A).

### CES-1 Snail Forges a Link between Asymmetric Cell Division and Apoptosis

Our results also indicate that the CES-1 protein acts in the NSM neuroblasts to affect the orientation of the cleavage plane and, hence, asymmetric cell division (Figure 6). Therefore, at least in the NSM lineage, the CES-1 protein represents a functional link between the cellular machinery that causes asymmetric cell division and the cellular machinery that causes the apoptotic death of specific cells during C. elegans development. At least to our knowledge, this is the first demonstration that apoptosis can directly be controlled by asymmetric cell division. Like the function of *ces-1* in NSM fate determination, the function of *ces-1* in asymmetric NSM neuroblast division is redundant to that of one or more unidentified genes. Similarly, the Snail-related proteins Snail, Escargot, and Worniu of D. melanogaster act redundantly to cause asymmetric cell division [14,15,17].

### DNJ-11 and CES-2 Regulate *ces-1* Transcription in the NSM Lineage

The HLF-like bZIP transcription factor, CES-2, is thought to act as a direct repressor of CES-1 Snail transcription [19,21]. We have found that the *dnj-11* MIDA1-like gene acts in concert with CES-2 as a negative regulator of CES-1 expression. Reducing either *dnj-11* or *ces-2* function results in an increased level of *ces-1* transcription within the NSM lineage, disrupts asymmetric NSM neuroblast division, and prevents the death of the NSM sister cells. Based on our data, *dnj-11* and *ces-2* could either act in parallel or in a single linear pathway to antagonize *ces-1* function (Figure 6). MIDA1-like proteins have been implicated in transcriptional regulation [28–30] and are components of a ribosome-associated chaperone referred to as RAC, which co-translationally interacts with nascent polypeptides thereby affecting translational accuracy and termination as well as protein folding [31,32]. The DNJ-11 protein predominantly localizes to the cytosol in a punctate pattern, which suggests that rather than regulating *ces-1* transcription directly, DNJ-11 might affect the translation and/or folding of a regulator of *ces-1* transcription.

### A Model for the Role of the *dnj-11* MIDA1, *ces-2* HLF, *ces-1* Snail Pathway in Asymmetric NSM Neuroblast Division

Based on our results, we propose that by repressing the transcription of the *snail*-related *ces-1* gene, the HLF-like bZIP transcription factor CES-2 and the MIDA1-like chaperone DNJ-11 ensure that the CES-1 protein is present at an appropriate, low level in the early NSM neuroblast (Figure 6A). A low level of CES-1 protein in the early NSM neuroblast would allow the expression at a certain level of a “polarity factor” that is required for the asymmetric division of the late NSM neuroblast. Furthermore, a complex that localizes to the ventral/medial side of the late NSM neuroblast would restrict the polarity factor to the ventral/medial side, thereby promoting the displacement of the mitotic spindle along the cell division axis and resulting in a shift of the cleavage plane and the asymmetric division of the cell. Finally, the asymmetrically localized polarity factor would also restrict CES-1 to the ventral/medial side and thereby cause its segregation predominantly into the NSM, thus resulting in the repression of *egl-1*/BH3-only transcription in the NSM and the survival of the NSM (Figure 6A). In this model, the CES-1 protein not only is a component of the cellular machinery that causes asymmetric NSM neuroblast division, but also one of its targets. The identity of the polarity factor as well as the signals and mechanisms that cause its asymmetric localization or activation remain to be determined. The Snail-related genes *snail*, *escargot*, and *worniu* of D. melanogaster function in asymmetric neuroblast division by promoting the expression of the gene *inscuteable*, which encodes an adaptor protein required for asymmetric neuroblast division. It will be of interest to determine whether the C. elegans ortholog of *inscuteable*, the gene *insc-1* (*F43E2.3*) (INSC-1 accession number AAC71125) [37], plays a role in asymmetric cell division and in asymmetric NSM neuroblast division, in particular.

*dnj-11(bc212)* does not affect asymmetric cell divisions that are known to be regulated by the C. elegans genes *ham-1* [4,38], *pig-1* [5], *dsh-2* [39], or *hlh-14* [40]. Conversely, the loss of *ham-1*, *pig-1*, *dsh-2*, or *hlh-14* function does not affect the asymmetric division of the NSM neuroblast. Therefore, the *dnj-11* MIDA1, *ces-2* HLF, and *ces-1* Snail pathway might be independent of these genes. Furthermore, the first division of the zygote, which occurs asymmetrically and is known to be regulated by the *C. elegans par* genes [41], is not affected by *dnj-11(bc212)*, *ces-2*(*bc213*), or *ces-1*(*n703*gf) (J. Hatzold, B. Conradt, unpublished data). Whether the *par* genes function in the asymmetric division of the NSM neuroblast remains to be determined.

### The Role of the *dnj-11* MIDA1, *ces-2* HLF, *ces-1* Snail Pathway in Asymmetric Cell Division and the Control of Apoptosis Might be Conserved

At least to our knowledge, this is the first evidence that a HLF-like bZIP transcription factor plays a role in asymmetric cell division. Furthermore, our studies provide a functional link between the known roles in asymmetric cell division of MIDA1-like chaperones and Snail-related transcription factors, and hence suggest the existence of a pathway involved in asymmetric cell division that is conserved throughout the plant and animal kingdoms [14–16,26]. Therefore, it will be of interest to determine whether HLF-like and Snail-related proteins also contribute to asymmetric cell division in V. carteri and whether MIDA1-like and HLF-like proteins also participate in asymmetric neuroblast division in D. melanogaster. Furthermore, our results hint at the possibility that MIDA1-like, HLF-like, and Snail-related proteins might play a role in asymmetric cell division in vertebrates. Specifically, it has recently been reported that HLF and the Snail-related protein Slug of mammals have functions in stem cells [12,42]. In addition, there is increasing evidence that the process of asymmetric cell division plays a crucial role in the ability of stem cells to self renew [43]. Therefore, we hypothesize that a *dnj-11* MIDA1, *ces-2* HLF, *ces-1* Snail–like pathway might be important for stem cell renewal by allowing asymmetric stem cell division.

At least to our knowledge, this is also the first evidence that a MIDA1-like protein plays a role in the regulation of apoptosis. Furthermore, our studies have identified a new component of a conserved cell-death specification pathway composed of *C. elegans ces-2* HLF and *ces-1* Snail. Like CES-2 and CES-1, HLF and the Snail-related protein Slug of mammals have previously been implicated in the regulation of apoptosis [19,21,44–46]. Therefore, it will be of interest to determine whether MIDA1-like proteins also have an apoptotic role in mammals and act in a *HLF-*, and *Slug*-dependent pathway. Finally, based on our work, we consider it a possibility that the roles in vertebrates of a *dnj-11* MIDA1, *ces-2* HLF, *ces-1* Snail–like pathway in asymmetric cell division and apoptosis might be functionally linked as well. Specifically, we speculate that such a pathway could cause stem cell renewal through asymmetric cell division and control stem cell numbers through apoptosis [47].

### Implications for the Functions of the Proto-Oncoprotein HLF and the Snail-Related Protein Slug of Mammals

The oncogenic form of human HLF, the E2A-HLF fusion protein, found in patients carrying the t(17;19) (q22;p13) translocation, gives rise to pro–B cell acute lymphoblastic leukemia (ALL) in adolescents [48]. The E2A-HLF fusion protein is composed of the *trans*-activation domain of the bHLH protein E2A and the DNA-binding domain of HLF [49]. It has been proposed that E2A-HLF causes leukemic transformation of pro-B cells by blocking their apoptotic death. Specifically, it has been proposed that E2A-HLF inappropriately activates the transcription of the *snail*-related gene, *slug*, which encodes a direct repressor of the *egl-1*-like, pro-apoptotic BH3-only gene *puma*, thereby causing the survival of pro-B cells that are normally programmed to undergo apoptosis [44–46]. Based on our results, we speculate that like C. elegans CES-2 and CES-1, the proteins HLF and Slug might not only function to control the expression of a pro-apoptotic BH3-only gene but to cause asymmetric cell division. Hence, in patients with the t(17;19) (q22;p13) translocation, the presence of the E2A-HLF fusion protein and elevated levels of Slug protein might affect aspects of the pro-B cell fate other than their apoptotic fate and/or might alter the division of the lymphoid progenitors that produce pro-B cells. Finally, it will be of interest to determine whether the human MIDA1-like gene MPP11 [27], which is expressed in hematopoietic lineages as well as other tissues, plays a role in E2A-HLF- and Slug-mediated tumorigenesis.

## Materials and Methods

### Strains and genetics.

C. elegans strains were cultured as described [50]. Bristol N2 was used as the wild-type strain. The strain CB4856 (Hawaii) was used in conjunction with N2 for SNP mapping. Mutations and transgenes used in this study are listed below and are described [51], except where noted otherwise: LGI: *ces-1(n703n1434)*, *ces-1(n703*gf*)*, *ces-2(n732*ts*)*, *ces-2(bc213)* (this study). LGII: *rrf-3(pk1426)* [52]. LGIII: *gmIs12* (P*_srb-6_gfp*) [53], *ced-4(n1162)*. LGIV: *unc-5(e53)*, *dnj-11(bc212*, *tm2859)* (this study), *dpy-20(e1282)*, *nDf41* [54], *bcIs25* (P*_tph-1_gfp*) [20]. LGV: *nIs83* (P*_mec-4_gfp*), *bcIs37* (P*_egl-1_gfp*) [20], *egl-1(n1084n3083)* [55]; *akIs3 (*P*_nmr-1_gfp)* [56]. (LGX) *nIs106* (P*_lin-11_gfp*) [57], *dtIs372* (M. Dunn, G. Seydoux, J. Waddle. personal communication). Additional stable transgenes used: *tIs38* (P*_pie-1_gfp*::*ph*(*PLC1δ1*)) [36], *kyIs39* (P*_sra-6_gfp*) [53], *bcIs58* (P*_ces-1_ces-1*::*yfp*) (this study). RNA-mediated interference (RNAi) by feeding was performed as described using 6 mM IPTG [58].

### Cloning of *dnj-11(bc212).*

Standard genetic techniques were used to map *dnj-11* between *unc-5* and *dpy-20* on LGIV. SNP mapping was used to locate *dnj-11* between the SNPs C43G2:22057 and C17H12:33927. The cosmid *F38A5* as well as a 3,565-bp subclone of *F38A5* contained in plasmid pBC484 (P*_dnj-11_dnj-11*) rescued the NSM sister cell survival and brood size phenotype observed in *bc212* animals (Table 2 and Table S5, *dnj-11(bc212)*; P*_dnj-11_dnj-11*). In addition, partially reducing *dnj-11* function by RNA-mediated interference (RNAi) causes 22% NSM sister cell survival (Table 1).

### Molecular analysis.

*dnj-11 plasmids:* pBC484 (P*_dnj-11_dnj-11*) was generated by inserting a *Eco*RV-*Pvu*I subclone of cosmid *F38A5* into the *Eco*RV site of pBluescript II KS + (Stratagene). The *gfp* sequence was amplified from pPD95.02 (gift of A. Fire, Stanford Scool of Medicine, Stanford, California) with appropriate primers (sequence of these and all other primers are available on request) and inserted into the *Bsm*I site of pBC484 to create a C terminal in frame fusion of *dnj-11* to *gfp* (P*_dnj-11_dnj-11*::*gfp*). The P*_dnj-11_dnj-11*::*gfp* transgene rescued the NSM sister cell survival phenotype of *dnj-11(bc212)* mutants, demonstrating that it is functional (Table 2, *dnj-11(bc212)*; P*_dnj-11_dnj-11*::*gfp*). To generate P*_dnj-11_dnj-11*::*gfp* mutant plasmids, site-directed PCR mutagenesis was performed to mutate CAC to CAA (H129Q), TGG to GGG (W456G), and TTC to GGC (F578G). The expression levels of the transgenes P*_dnj-11_dnj-11*(H129Q)::*gfp*, P*_dnj-11_dnj-11*(W456G)::*gfp*, P*_dnj-11_dnj-11*(F578G)::*gfp*, and P*_dnj-11_dnj-11*(W456G F578G)::*gfp* were similar to that of the wild-type transgene (P*_dnj-11_dnj-11*::*gfp*), and the subcellular localization of the resulting fusion proteins was indistinguishable from that of DNJ-11::GFP (unpublished data).

*ces-1 plasmids:* The plasmid pBC510 (P*_ces-1_ces-1*::*yfp*) was generated by cloning an *Afl*II-*Spe*I fragment from cosmid *F43G9* containing the *ces-1* rescuing fragment [19] into the EcoRV site of pBluescript II KS + (pBC482A), and inserting *yfp* amplified from pvdB#3 [59] into the *Swa*I site to generate a C terminal in frame fusion. The P*_ces-1_ces-1*::*yfp* transgene was able to block the ability of *ces-1(n703n1434)* to suppress the NSM sister cell survival phenotype of *dnj-11(bc212)* and *ces-2(n732)* animals, demonstrating that it is functional (unpublished data). To generate plasmid pBC664 (P*_ces-1_gfp*), first *ces-1* upstream and downstream regulatory regions were amplified by PCR, a *Pme*I site was introduced 3′ of upstream and 5′ of downstream regulatory regions, and PCR products were cloned into *Xma*I/*Nco*I digested pBC482A to obtain the *ces-1* locus without coding region, 5′ and 3′ untranslated regions (UTRs), and introns (pBC656). Because intron 4 of *ces-1* is highly conserved between C. elegans and C. briggsae, intron 1 of the *gfp* sequence of plasmid pPD95.77 (gift of A. Fire) was replaced with intron 4 of *ces-1* by PCR fusion. The *Sma*I/*Spe*I fragment of pPD95.77_*ces-1* intron containing the *gfp* sequence and the *unc-54* 3′ UTR was inserted into the *Pme*I site of pBC656 to generate pBC664.

### Transgenic animals.

Germline transformation was performed as described [60]. Cosmids were injected at a concentration of 10 ng/μl with pPD93.97 (P*_myo-3_gfp*) at 50 ng/μl as coinjection marker. Plasmids were injected at a concentration of 10 ng/μl with pRF4 (*rol-6(su1006)*) at 50 ng/μl as coinjection marker. pBC510 (P*_ces-1_ces-1*::*yfp*) was injected into N2 to create an extrachromosomal array and integrated using EMS mutagenesis [50] to generate *bcIs58*. The strain carrying *bcIs58* was backcrossed three times to N2.

### Phenotypic analysis and microscopy.

The NSM sister cell survival was scored as previously described [20]. Microscopy of living embryos was performed by mounting embryos on 2%–5% agar pads in M9 buffer, sealing them with petroleum jelly, and using a Zeiss Axioskop2 equipped with epifluorescence, a Micromax CCD camera (Princeton Instruments), and Metamorph software. NSM neuroblasts, NSMs, and NSM sister cells were identified based on the position of their nuclei using Nomarski optics. Z-series were taken with a Z-distance of 0.5 μm (analysis of P*_ces-1_ces-1*::*yfp*, P*_ces-1_gfp*, and P*_his-24_his-24*::*gfp* expression) and 0.25 μm (determination of cell size). Epifluorescence Z-series were deconvolved using the AutoDeblur Gold WF AutoVisualize software (Media Cybernetics). The cell size of NSMs and NSM sister cells was determined 10 to 15 min after the NSM neuroblast had started to divide, as indicated by the breakdown of the nuclear envelop, which was observed by Nomarski optics. To visualize the outline of a cell, a plasma membrane–targeted GFP fusion protein (P*_pie-1_gfp*::*ph*(*PLC1*δ*1*)) was used. The area of the cross section of a cell was measured in each section of a Z-series using Metamorph software. To estimate the difference in volume between NSM and NSM sister cell, the values of each section were added, and the sum obtained for the NSM sister cell was divided by the sum obtained for the NSM. To determine the orientation of the cleavage plane of the NSM neuroblast, chromatids were visualized using a His24-GFP fusion protein (P*_his-24_his-24*::*gfp*, *dtIs372*) (gift of M. Dunn and G. Seydoux, Johns Hopkins University, Baltimore, Maryland; and J. Waddle, Southern Methodist University, Dallas, TX). The NSM neuroblast was identified by Nomarski optics before the start of the division and consecutive Z series were taken in 1 min time intervals until the completion of the NSM neuroblast division.

### Immunohistochemistry.

Embryos were prepared in 10 μl on poly L-lysine coated slides and fixed and stained as described [61]. Slides were mounted in 1 μg/ml DAPI in PBS 1:1 diluted with VectaShield (Vector Laboratories). GFP was detected using the anti-AFP antibody monoclonal antibody 3E6 (Qbiogene).

## Supporting Information

Figure S1 *dnj-11(bc212)* Animals Display Various Morphological Defects (A) Nomarski images of wild-type (+/+) and *dnj-11(bc212)* embryos at the 1.5- and 2-fold stage, respectively. Note that *dnj-11(bc212)* embryos show morphological defects.
(B and C) Nomarski images of wild-type (+/+) and *dnj-11(bc212)* larvae. Arrows point to mis-shaped structures.
(D) Nomarski images of the vulva region of wild-type (+/+) and *dnj-11(bc212)* adults. The arrow points to a defective vulva, the arrowhead points to a distorted body area. The complete genotypes of the analyzed animals were: *bcIs25* and *dnj-11(bc212) bcIs25*.
(796.79 KB).

Table S1 *dnj-11(bc212)* Is Maternally Rescued The NSM sister cell survival was scored as described in Materials and Methods. Strains were grown and analyzed at 20 °C. The complete genotype of the strains analyzed was *bcIs25*, *dnj-11(bc212) bcIs25*, *unc-5(e53) dpy-20(e1282) bcIs25 / dnj-11(bc212) bcIs25*, *unc-5(e53) dnj-11(bc212) dpy-20(e1282) bcIs25*, *dnj-11(bc212) bcIs25*; *bcEx513 (*P*_dnj-11_dnj-11)*, and *dnj-11(bc212) bcIs25*. The complete maternal genotype of the strains analyzed was *bcIs25*, *dnj-11(bc212) bcIs25*, *unc-5(e53) dpy-20(e1282) bcIs25*, *unc-5(e53) dnj-11(bc212) dpy-20(e1282) bcIs25 / bcIs25*, *dnj-11(bc212) bcIs25*; *bcEx513* (P*_dnj-11_dnj-11*), and *dnj-11(bc212) bcIs25*; *bcEx513* (P*_dnj-11_dnj-11*).
(33.50 KB ).

Table S2 *dnj-11(bc212)* Does Not Cause a General Cell Death Defect The presence of surviving cells in the anterior pharynx was scored by Nomarski Optics as described [55,62]. The presence of surviving cells in the ventral cord was scored using the reporter P*_lin-11_gfp* [57]. The presence of surviving PVQ sister cells was determined using P*_sra-6_gfp* [53]. The presence of extra PHB cells was scored using P*_srb-6_gfp* [53]. All strains were raised and analyzed at 15 °C. The complete genotype of the strains analyzed was *bcIs25* (P*_tph-1_gfp*) (+/+) and *dnj-11(bc212) bcIs25* (*dnj-11(bc212)*), *nIs106* (P*_lin-11_gfp*) (+/+) and *dnj-11(bc212) bcIs25*; *nIs106* (*dnj-11(bc212)*), *kyIs39* (P*_sra-6_gfp*) (+/+) and *dnj-11(bc212) bcIs25*; *kyIs39* (*dnj-11(bc212)*), and *gmIs12* (P*_srb-6_gfp*) (+/+) and *dnj-11(bc212) bcIs25*; *gmIs12* (*dnj-11(bc212)*).
(56.50 KB).

Table S3 *dnj-11(bc212)* and *dnj-11(tm2859)* Cause Embryonic Lethality Embryonic lethality was analyzed by allowing adults of genotype *bcIs25* (+/+) or *dnj-11(bc212) bcIs25* (*dnj-11(bc212)*) or *bcIs30* and dnj-11(tm2859)*; *bcIs30* to lay eggs for 3 h. Adults were removed and eggs laid during the 3-h period were counted. Unhatched eggs were scored after 1, 2, and 3 d. The animals were raised and the experiment performed at 15 °C.
(25.50 KB).

Table S4 *dnj-11(bc212)* Mutants Grow Slower than Wild Type Strains were grown and analyzed at 15 °C. The growth rate was analyzed by allowing five adults of genotype *bcIs25* (+/+) or *dnj-11(bc212) bcIs25* (*dnj-11(bc212)*) to lay eggs for 3 h. Adults were removed and the number of eggs laid during the 3-h period was determined. The presence of L4 larvae was monitored daily starting at day 5.
(27.00 KB ).

Table S5 *dnj-11(bc212)* Causes a Reduced Brood Size. Animals were grown and analyzed at 15 °C. Individual L4 larvae were plated onto plates and transferred to fresh plates daily for 4–5 d. The total number of progeny laid during the 4–5-d period was determined by counting the presence of L4 larvae. The standard deviation (STDEV) of the brood size of five different animals is indicated. All animals analyzed were homozygous for the integration *bcIs25* (P*_tph-1_gfp*).
(28.00 KB).

Table S6 *dnj-11(bc212)* Causes a Block of *egl-1* Expression in the NSM Sister Cells The presence of GFP in the NSM sister cells was scored in L1 larvae using Nomarski optics and the integrated *egl-1* reporter *bcIs37* (P*_egl-1_his24-gfp*). Animals were grown at 15 °C.
(29.00 KB ).

Table S7 *dnj-11(bc212)* Is Not Required for Establishing Cell Polarity in General The asymmetric division of cells indicated in the left column was assayed by scoring missing or extra cells generated by their respective lineage. Missing or extra P*_srb-6_gfp–*expressing PHB neurons were scored to analyze the asymmetric division of ABpl/rapppap (the disruption of the asymmetry of this division by mutations in *ham-1* results in missing or extra P*_srb-6_gfp* expressing PHB neurons [38)]. Missing or extra P*_mec-4_gfp–*expressing PLM neurons were scored to analyze the asymmetric division of ABpl/rapapppp (the disruption of the asymmetry of this division by mutations in *ham-1* or *pig-1* results in missing or extra P*_mec-4_gfp* expressing PLM neurons [5,38]). Missing or extra I2 neurons were scored by Nomarski optics to analyze the asymmetric division of ABalpappaa/ABarapapaa (the disruption of the asymmetry of this division by mutations in *ham-1* or *pig-1* results in extra I2 neurons [4,5,38]). Missing or extra M4 neurons were scored by Nomarski optics to analyze the asymmetric division of MSpaaaaa (the disruption of the asymmetry of this division by mutations in *pig-1* results in extra I2 neurons [5]). Missing or extra P*_mec-4_gfp–*expressing A/PVM neurons were scored to analyze the asymmetric division of QR/L.p (the disruption of the asymmetry of this division by mutations in *pig-1* results in extra A/PVM neurons [5]). Missing or extra P*_nmr-1_gfp–*expressing PVC neurons were scored to analyze the asymmetric division of ABpl/rpppa (the disruption of the asymmetry of this division by mutations in *dsh-2* results in extra PVC neurons [39]). Missing or extra P*_sra-6_gfp–*expressing PVQ neurons were scored to analyze the asymmetric division of ABpl/rappp (the disruption of the asymmetry of this division by mutations in *hlh-14* results in missing PVQ neurons [40]).
(86.00 KB).

### Accession Numbers

The NCBI Entrez Protein (http://www.ncbi.nlm.nih.gov/sites/entrez?db=protein) accession numbers for genes and gene products discussed in this paper are: NP_501006 (DNJ-11), NP_506575 (EGL-1), NP_492338 (CES-1), NP_493610 (CES-2), NP_033610 (mouse MIDA1), NP_055192 (human MPP11), NP_011801 (S. cerevisiae Zuotin).

The GenBank (http://www.ncbi.nlm.nih.gov/) accession number for V. carteri GlsA is AF_106963.

## Abbreviations

aa: amino acid
CES: cell-death specification
CED: cell-death abnormal
DNJ: DnaJ domain
Egl: egg-laying abnormal
GFP: green fluorescent protein
Gls: gonidialess
HLF: hepatic leukemia factor
Insc: Inscuteable (*Drosophila* asymmetric cell division protein) homolog
LG: linkage group
MIDA: mouse Id associated
MPP: M phase phosphoprotein
NSM: neurosecretory motoneuron
