# Supplementary material for: Control of Apoptosis by Asymmetric Cell Division
Source: PLoS Biol. 2008 Apr 8;6(4):e84. doi: 10.1371/journal.pbio.0060084 (PMC2288629; doi:10.1371/journal.pbio.0060084)
Supplement: Supplementary file 1 [file pbio.0060084.st001.doc]

Table S1. *dnj-11(bc212)* is maternally rescued.

| Genotype | Maternal Genotype | NSM sister cell survival [%] (n) | |
| --- | --- | --- | --- |
| +/+ | +/+ | 0 | (414) |
| *dnj-11(bc212)* | *dnj-11(bc212)* | 28 | (214) |
| *dnj-11(bc212) / +* | +/+ | 0 | (70) |
| *dnj-11(bc212)* | *dnj-11(bc212)* / *+* | 1 | (136) |
| *dnj-11(bc212);* P*_dnj-11_dnj-11* | *dnj-11(bc212);* P*_dnj-11_dnj-11* | 3 | (98) |
| *dnj-11(bc212)* | *dnj-11(bc212);* P*_dnj-11_dnj-11* | 1 | (74) |
|  | | | |
