# Supplementary material for: Control of Apoptosis by Asymmetric Cell Division
Source: PLoS Biol. 2008 Apr 8;6(4):e84. doi: 10.1371/journal.pbio.0060084 (PMC2288629; doi:10.1371/journal.pbio.0060084)
Supplement: Supplementary file 2 [file pbio.0060084.st002.doc]

Table S2. *dnj-11(bc212)* does not cause a general cell death defect

| Cell destined to die |  | Surviving cells [%] (n) | | | |
| --- | --- | --- | --- | --- | --- |
|  |  | +/+ | | *dnj-11(bc212)* | |
| **Anterior pharynx** |  |  |  |  |  |
| NSM sister | L/R | 0 | (19) | 45 | (19) |
| m2 sister | L/R | 0 | (19) | 0 | (19) |
| e1 sister |  | 0 | (19) | 0 | (19) |
| I1 sister | L/R | 0 | (19) | 0 | (19) |
| m1 sister | VL/VR | 0 | (19) | 0 | (19) |
| I2 sister | L/R | 3 | (19) | 5 | (19) |
| MCL sister |  | 0 | (19) | 0 | (19) |
| M4 sister |  | 0 | (19) | 0 | (19) |
| sister of e1 precursor |  | 0 | (19) | 0 | (19) |
| sister of I2 precursor | L/R | 0 | (19) | 0 | (19) |
| **Ventral cord** |  |  |  |  |  |
| P1aap |  | 0 | (33) | 0 | (25) |
| P2aap |  | 0 | (33) | 0 | (25) |
| P9aap |  | 0 | (33) | 0 | (25) |
| P10aap |  | 0 | (33) | 0 | (25) |
| P11aap |  | 0 | (33) | 0 | (25) |
| P12aap |  | 0 | (33) | 0 | (25) |
| **Additional cells** |  |  |  |  |  |
| PVQ sister | L/R | 0 | (50) | 0 | (35) |
| sister of HSN/PHB precursor | L/R | 0 | (30) | 0 | (27) |
