# Supplementary material for: Control of Apoptosis by Asymmetric Cell Division
Source: PLoS Biol. 2008 Apr 8;6(4):e84. doi: 10.1371/journal.pbio.0060084 (PMC2288629; doi:10.1371/journal.pbio.0060084)
Supplement: Supplementary file 3 [file pbio.0060084.st003.doc]

Table S3. *dnj-11(bc212)* and *dnj-11(tm2859)* cause embryonic lethality.

| Genotype | Unhatched eggs [% of laid eggs] (n) | |
| --- | --- | --- |
| +/+ | 2 | (122) |
| *dnj-11(bc212)* | 24 | (108) |
| +/+* | 2 | (222) |
| *dnj-11(tm2859)** | 26 | (47) |
