# Supplementary material for: Control of Apoptosis by Asymmetric Cell Division
Source: PLoS Biol. 2008 Apr 8;6(4):e84. doi: 10.1371/journal.pbio.0060084 (PMC2288629; doi:10.1371/journal.pbio.0060084)
Supplement: Supplementary file 4 [file pbio.0060084.st004.doc]

Table S4. *dnj-11(bc212)* mutants grow slower than wild type.

|  | L4s [% of laid eggs] | | | | | |
| --- | --- | --- | --- | --- | --- | --- |
| Genotype | 5 days | 5 ½ days | 6 days | 6 ½ days | 7 days | 8 days |
| +/+ | 70 | 83 | 89 | 90 | 91 | 92 |
| *dnj-11(bc212)* | 0 | 0 | 13 | 38 | 52 | 54 |
