# Supplementary material for: Control of Apoptosis by Asymmetric Cell Division
Source: PLoS Biol. 2008 Apr 8;6(4):e84. doi: 10.1371/journal.pbio.0060084 (PMC2288629; doi:10.1371/journal.pbio.0060084)
Supplement: Supplementary file 5 [file pbio.0060084.st005.doc]

Table S5. *dnj-11(bc212)* causes a reduced brood size

| Genotype | Brood size | STDEV | n |
| --- | --- | --- | --- |
| +/+ | 231 | 39 | 10 |
| *dnj-11(bc212)* | 52 | 13 | 10 |
| *dnj-11(bc212)*; P*_dnj-11_dnj-11* | 199 | 48 | 8 |
| *ces-1(n703n1434)* | 193 | 37 | 10 |
| *ces-1(n703n1434)*; *dnj-11(bc212)* | 36 | 20 | 9 |
