# Supplementary material for: Control of Apoptosis by Asymmetric Cell Division
Source: PLoS Biol. 2008 Apr 8;6(4):e84. doi: 10.1371/journal.pbio.0060084 (PMC2288629; doi:10.1371/journal.pbio.0060084)
Supplement: Supplementary file 6 [file pbio.0060084.st006.doc]

**Table S6. *dnj-11(bc212)* causes a block of *egl-1* expression in the NSM sister cells**

| Genotype | *gfp* expressing NSM sister cells [%] (n) | |
| --- | --- | --- |
| *ced-4(n1162); bcIs37* | 100 | (15) |
| *ced-4(n1162); unc-5(e53) dnj-11(bc212); bcIs37* | 31 | (17) |
