# Supplementary material for: Control of Apoptosis by Asymmetric Cell Division
Source: PLoS Biol. 2008 Apr 8;6(4):e84. doi: 10.1371/journal.pbio.0060084 (PMC2288629; doi:10.1371/journal.pbio.0060084)
Supplement: Supplementary file 7 [file pbio.0060084.st007.doc]

Table S7. *dnj-11(bc212)* is not required for establishing cell polarity in general

| Cell division | Cells analyzed  (Reporter used) | Genotype | [%] Cells | | n |
| --- | --- | --- | --- | --- | --- |
|  |  |  | Missing | Extra |  |
| ABpl/rapppap  **PHB**  X  HSN | PHBL/R |  |  |  |  |
|  | (P*_srb-6_gfp*) | wt | 0 | 0 | 30 |
|  |  | *dnj-11(bc212)* | 0 | 0 | 27 |
| ALN  X  **PLM**  ABpl/rapapppp | PLML/R |  |  |  |  |
|  | (P*_mec-4_gfp*) | wt | 2 | 0 | 65 |
|  |  | *dnj-11(bc212)* | 1 | 0 | 73 |
| ABalpappaa/ABarapapaa  X  **I2**  X | I2L/R |  |  |  |  |
|  |  | wt | 0 | 3 | 19 |
|  |  | *dnj-11(bc212)* | 0 | 5 | 19 |
| **M4**  X  MSpaaaaa | M4 |  |  |  |  |
|  |  | wt | 0 | 0 | 19 |
|  |  | *dnj-11(bc212)* | 0 | 0 | 19 |
| X  SDQL/R  **A/PVM**  QR/L.p | A/PVM |  |  |  |  |
|  | (P*_mec-4_gfp*) | wt | 8 | 0 | 65 |
|  |  | *dnj-11(bc212)* | 9 | 1 | 45 |
|   ABpl/rpppa | PVCL/R |  |  |  |  |
|  | (P*_nmr-1_gfp*) | wt | 0 | 0 | 11 |
|  |  | *dnj-11(bc212)* | 0 | 0 | 24 |
|   ABpl/rappp | PVQR/L |  |  |  |  |
|  | (P*_sra-6_gfp*) | wt | 0 | 0 | 50 |
|  |  | *dnj-11(bc212)* | 1 | 0 | 34 |
